# The complete mitochondrial genome and the phylogenetic position of *Alauda gulgula* (Aves: Passeriformes)

**DOI:** 10.1080/23802359.2021.1909438

**Published:** 2021-04-08

**Authors:** Shangmingyu Zhang, Xiaofeng Zheng, Chuang Zhou, Min Zhu, Yongjie Wu

**Affiliations:** a Key Laboratory of Bio‐resources and Eco‐environment, Ministry of Education, College of Life Sciences, Sichuan University, Chengdu, China; b Sichuan Key Laboratory of Conservation Biology on Endangered Wildlife, College of Life Sciences, Sichuan University, Chengdu, China; c The General Work Station of Protected Area of Sichuan Province, Chengdu, China

**Keywords:** *Alauda gulgula*, mitochondrial genome, phylogenetic analysis

## Abstract

The Oriental Skylark (*Alauda gulgula*) is a small songbird in the Alaudidae. Here we assembled the complete mitochondrial genome of *Alauda gulgula* which is 17,055 bp in length and consisting of 13 protein-coding genes (PCGs), 2 ribosomal RNA, 22 transfer RNA, and 2 extensive heteroplasmic control regions. The overall A + T content of the mitogenome is 52.3%The maximum-likelihood (ML) tree based on the complete mitochondrial genome of *A. gulgula* revealed the close genetic relationship between *A. gulgula* and *A. arvensis,* but separate from *A. cheleensis*.

The Oriental Skylark (*Alauda gulgula*) is a common frugivore and insectivore distributed in the palearctic with small erectile crest. Larks are difficult to delineate based on morphology, because of their similar plumage pattern (Alström et al. 2013). Morpho-anatomy is not reliable for phylogenetic assessment (Ericson and Johansson 2003).Clarifying the phylogenetic relationships based on complete mitochondrial sequences could contribute to the study of the phylogenetic relationships in the Alaudidae (Zhou et al. 2009), as well as a reassessment of lark relationships and taxonomy.

Muscle tissue of a wild *A. gulgula* collected from an airport protection facility was gathered from Ganzi Gesser Airport (99.553986E, 31.756744 N) in July 2020. The specimen was deposited in the Natural Museum of Sichuan University under the voucher number of QZKK088 (Curator name: Jianghong Ran; Email: rjhong-01@163.com). The total genomic DNA was extracted using the M5 HiPer Universal DNA Mini Kit following the manufacturer’s instructions. The mitogenome of *A. gulgula* were generated by amplification of overlapping Polymerase Chain Reaction (PCR) fragments. The thirteen fragments were amplified using the universal primers (Amer et al. 2013) following the instructions of the 2× Rapid Taq Master Mix (Vazyme Biotech Co., Ltd). Sequences obtained were aligned and annotated using the software SeqMan (DNAStar, Inc.) and MITOS Web Server (Bernt et al. 2013), respectively.

The complete mitogenome of *Alauda gulgula* (GenBank accession number MW304005) was 17,055 bp in length and consisted of 13 protein-coding (PCGs), 2 ribosomal RNA (rRNA), 22 transfer RNA (tRNA), and 2 extensive heteroplasmic control regions, which is consistent with other vertebrate mitogenomes (Qian et al. 2013). The majority of genes are encoded on the H-strand, however ND6 and eight tRNAs (tRNA^Gln^, tRNA^Ala^, tRNA^Asn^, tRNA^Cys^, tRNA^Tyr^, tRNA^Ser(UCN)^, tRNA^Pro^ and tRNA^Glu^) are encoded on the L-strand. The overall base composition of the *A. gulgula* mitogenome was 29.3% A, 33.0% C, 14.7% G, and 23.1% T with an A + T content of 52.3%. The AT skew was calculated as 0.12.

To further understand the evolutionary history of *A. gulgula*, PhyloSuite (Zhang et al. 2020) was used to construct a maximum-likelihood (ML) phylogenetic tree on the basis of the 12 mitochondrial protein-coding genes (except ND6 gene). TVM + F+G4 was selected as the substitution model according to the Bayesian Information Criterion (BIC) test based on Modeltest. Phylogenetic analysis of the mitogenome of *A. gulgula* resolved it on a fully supported branch with *A. arvensis* (Figure 1). These findings are consistent with the results of a previous study (Alström et al. 2013). In conclusion, this study confirmed the phylogenetic placement of *A. gulgula* and contributes to further investigations on the molecular evolution and conservation of this species.

**Figure 1.**
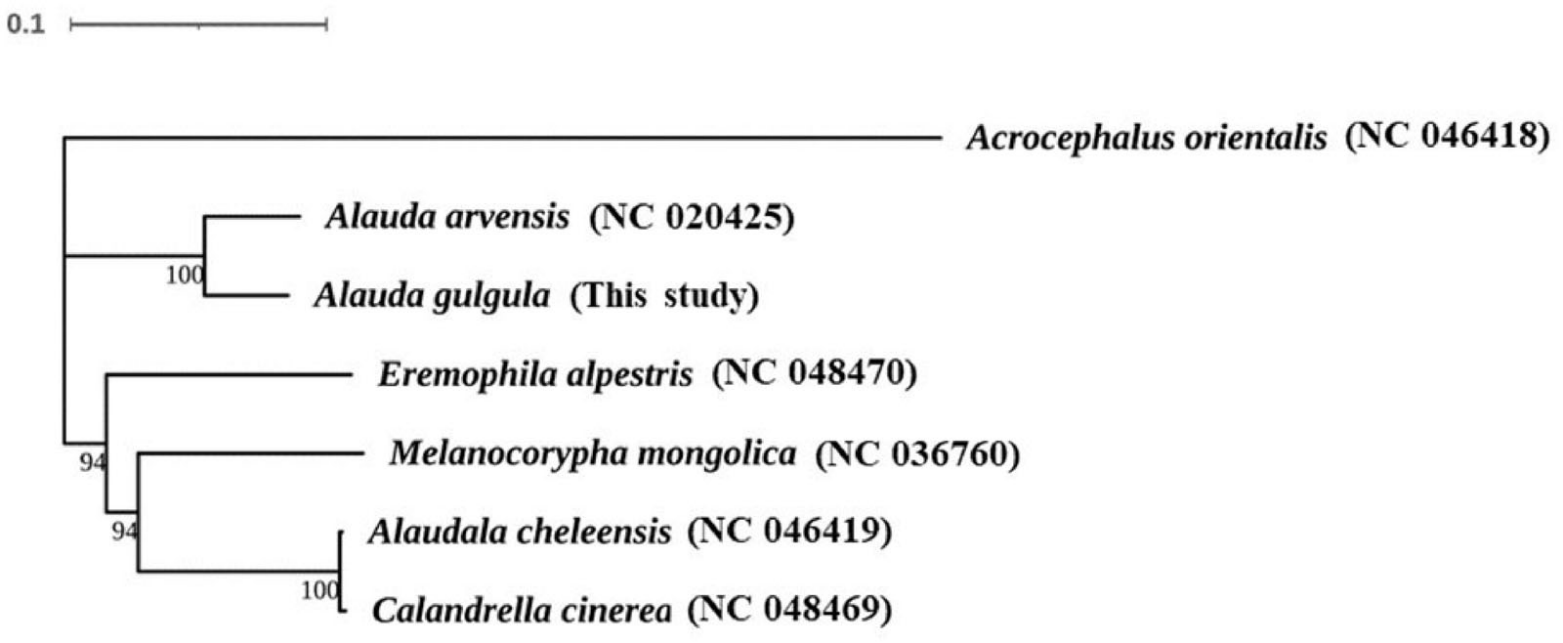
Phylogenetic tree of *Alauda gulgula* based on the maximum likelihood (ML) analysis of 12 concatenated mitochondrial protein-coding genes. *Acrocephalus orientalis* was designated as the outgroup. The bootstrap values are based on 5000 replicates and shown at the nodes.

## Data Availability

The data are openly available in GenBank of NCBI at https://www.ncbi.nlm.nih.gov, reference number MW304005.
